# Perceptual misalignment of texture representations in convolutional neural networks

**DOI:** 10.1167/jov.26.9.9

**Published:** 2026-09-18

**Authors:** Ludovica de Paolis, Fabio Anselmi, Alessio Ansuini, Eugenio Piasini

**Affiliations:** 1 Neuroscience Area, International School for Advanced Studies (SISSA), Trieste, Italy; 2 Department of Mathematics, Informatics and Geosciences, Università degli Studi di Trieste, Trieste, Italy; 3 Department of Data Engineering, Area Science Park, Trieste, Italy

**Keywords:** textures, convolutional neural networks, Brain-Score, representational similarity analysis

## Abstract

Mathematical modeling of visual textures traces back to Julesz’s intuition that texture perception in humans is based on local correlations between image features. An influential approach for texture analysis and generation generalizes this notion to linear correlations between the nonlinear features computed by convolutional neural networks (CNNs), compiled into Gram matrices. Given that CNNs are often used as models for the visual system, it is natural to ask whether such “texture representations” spontaneously align with the textures’ perceptual content, and, in particular, whether those CNNs that are regarded as better models for the visual system also possess more human-like texture representations. Here we quantify the perceptual content captured by feature correlations computed for a diverse pool of CNNs, and we compare it to the models’ perceptual alignment with the mammalian visual system as measured by Brain-Score. Surprisingly, we find that there is no connection between conventional measures of CNN quality as a model of the visual system and its alignment with human texture perception. We conclude that texture perception involves mechanisms that are distinct from those that are commonly modeled using approaches based on CNNs trained on object recognition, possibly depending on the integration of contextual information.

## Introduction

Visual textures hold a special status in vision neuroscience as stimuli that afford a nontrivial degree of controllability and complexity, unlike natural images or more traditional parametric stimuli (Victor, Conte, & Chubb, 2017). Because of this, multiple mathematical frameworks for modeling and generating textures have been established, with considerable success. One particularly influential strand of theoretical, computational, and empirical work stems from the seminal view of Julesz (Julesz, 1962; Caelli & Julesz, 1978; Julesz, 1981), who proposed that texture perception is controlled by local correlations of visual features within an image. This view was extended by later work that better formalized the notion of textures as statistical objects (Zhu, Liu, & Wu, 2000; but see Liu et al., 2019, for a detailed historical overview). This study demonstrated the effectiveness of using features based on the neuroscience of vision (Portilla & Simoncelli, 2000) and developed a connection with natural stimulus statistics through the efficient coding paradigm (Hermundstad et al., 2014; Tesileanu et al., 2020; Tesileanu, Piasini, & Balasubramanian, 2022; Tkačik, Prentice, Victor, & Balasubramanian, 2010; Victor & Conte, 2012; Victor et al., 2017; Zanon et al., 2026; Caramellino et al., 2021).

In recent years, following the rise in popularity of deep convolutional neural networks (CNNs) as tools in computer vision, Gatys et al. proposed a machine learning–based operationalization of Julesz’s idea (Gatys, Ecker, & Bethge, 2015). The Gatys model effectively defined a texture ensemble based on the Gram matrices formed by spatial correlations between intermediate-layer activations of a CNN trained for object recognition. Texture synthesis is performed by optimizing the image pixels via gradient descent to match the statistical structure of an exemplar texture. This is achieved by minimizing a *Gram loss*, defined as the normalized L2 distance between the Gram matrices of the exemplar image and those of the synthesized image. This approach was rapidly adopted in the budding fields of neural texture synthesis and style transfer (see e.g., Gatys, Ecker, & Bethge, 2016; Johnson, Alahi, & Fei-Fei, 2016; Li et al., 2017; Ulyanov, Vedaldi, & Lempitsky, 2017; Yu, Barnes, Shechtman, Amirghodsi, & Lukac, 2019), and was later improved under multiple aspects: high-resolution image processing (Snelgrove, 2017), better methods for capturing long-range correlations within images (Berger & Memisevic, 2017; Liu, Gousseau, & Xia, 2016; Sendik & Cohen-Or, 2017), and usage of more sophisticated losses capable of capturing stationary properties of the CNN’s activations beyond the pairwise correlations in the Gram loss (Heitz, Vanhoey, Chambon, & Belcour, 2021; Vacher, Davila, Kohn, & Coen-Cagli, 2020).

In parallel, and more broadly, CNNs trained for object recognition have become ubiquitous tools in vision neuroscience. Over the years, work from many labs has established these models as functional accounts of the ventral visual stream in monkeys (Bashivan, Kar, & DiCarlo, 2019; Cadena et al., 2019a; Cadieu et al., 2014; Khaligh-Razavi & Kriegeskorte, 2014; Schrimpf et al., 2020; Yamins, Hong, Cadieu, Solomon, & DiCarlo, 2014) and humans (Güçlü & Gerven, 2015; Storrs, Kietzmann, Walther, Mehrer, & Kriegeskorte, 2021), as well as the corresponding visual cortical areas in rodents (Cadena et al., 2019b; Muratore, Tafazoli, Piasini, Laio, & Zoccolan, 2022; Muratore, Alemi, & Zoccolan, 2025; Nayebi et al., 2023). However, the strong predictive power of this approach for neural activity is only partly mirrored by the capacity of these artificial neural networks to accurately predict behavioral responses to individual stimuli (Feather, Leclerc, Ma̧dry, & McDermott, 2023; Geirhos et al., 2021; Rajalingham et al., 2018; Vinken, Boix, & Kreiman, 2020; Wichmann & Geirhos, 2023).

Beyond object recognition, the ventral stream is also thought to be important for texture perception. Indeed, the idea that texture perception can be explained by reference to the neural mechanisms in early vision goes at least as far back as Turner (1986) and Malik and Perona (1990). More recent results in computational modeling and neurophysiological evidence point generally to the involvement of early-to-intermediate areas in the ventral stream (Freeman & Simoncelli, 2011; Freeman, Ziemba, Heeger, Simoncelli, & Movshon, 2013; Henderson, Tarr, & Wehbe, 2023; Matteucci, Piasini, & Zoccolan, 2024; Okazawa, Tajima, & Komatsu, 2017; Portilla & Simoncelli, 2000; Yu, Schmid, & Victor, 2015; Ziemba, Freeman, Movshon, & Simoncelli, 2016; Ziemba et al., 2024). However, overall, our understanding of the neural computations underpinning texture perception is still limited compared to those enabling object recognition. This makes CNN-based methods for texture analysis and synthesis particularly interesting from a neuroscience perspective, despite the recent emergence of approaches based on diffusion models for practical applications (e.g., Wang, Holynski, Curless, & Seitz, 2024). More specifically, it raises the question of whether the existing quality measures devised for CNN-based models of the ventral stream align with the perceptual quality of textures generated based on the internal features of those models. If such alignment exists, it would not only suggest that object recognition and texture perception share a significant amount of functional mechanisms, but also that these mechanisms are among those that are well captured by CNNs trained on object recognition. Conversely, the absence of such a relationship — where increased fidelity to neural or behavioral object recognition data does not lead to improved modeling of human texture perception — would indicate that current CNN architectures or training paradigms lack critical components.

In the present work, we considered a set of popular CNNs and asked whether the models that are more “brain-like” in object recognition also tend to represent visual textures in a way that is closer to human perception. As a metric of model performance on object recognition, we used the standardized benchmarks provided by Brain-Score (Schrimpf et al., 2018). To measure alignment with human texture perception, we evaluated the models on the images of the Describable Texture Dataset (Cimpoi, Maji, Kokkinos, Mohamed, & Vedaldi, 2014), which are divided into classes according to human annotations. We then assessed whether the “texture representations” provided by the Gram matrices computed from the models’ feature maps (as in Gatys et al., 2015) are organized in a way that respect the classes subdivision in the dataset.

Our results show that there is no correlation between the goodness of the Gram texture representation — with respect to the clusters formed by human-annotated labels — and Brain-Score. This suggests that the features governing the utility of artificial neural networks as models of visual cortex in an object recognition context are not fully aligned with those that can make them good models for texture processing, therefore highlighting an underexplored path for the improvement of machine learning–based models for texture analysis and synthesis.

## Methods

### The “Gram representation” for textures

Our study builds on the texture analysis and synthesis method introduced by (Gatys et al., 2015). This approach models textures using feature representations extracted from a CNN, which are shown to support the generation of perceptually compelling texture samples. In the original implementation, given a natural texture image *x*, the algorithm starts by feeding *x* as input to the network and extracting the corresponding activation maps from a predetermined set of layers. For each selected layer *l*, the feature activations *F*^*l*^ are used to compute a Gram matrix *G*^*l*^, defined as the inner product between vector representations of the feature maps:

(1) $$\begin{array}{c} G_{ij}^{l}=\sum_{m}F_{im}^{l}F_{jm}^{l},\; \end{array}$$

where the index *m* runs over spatial samples of the feature maps, and *i* and *j* label feature channels at layer *l*. This Gram matrix summarizes the correlations between feature channels, discarding spatial information and yielding a statistical description of textures. These “Gram representations” are thought to capture the elements of the input image that are important for texture perception. In this work, we study the Gram representations generated by a number of different CNNs.

### Texture synthesis

In the present, work we provide examples of texture synthesis to help the reader gauge the quality of the images that can be produced based on the representations described in the previous paragraph, despite the fact that our main analyses and conclusions do not depend on the output of any texture synthesis algorithm. For completeness, we report here the details of our synthesis approach.

To generate synthetic textures, we followed the method established by Gatys et al. (2015), with minor modifications. Briefly, texture synthesis is performed by iteratively updating the pixels of an initially random image $\hat{x}$, where pixel values are drawn from a Gaussian distribution $\hat{x}∼N\left(0,1\right)$. This process leads to matching the Gram matrices of $\hat{x}$ to those of the target image *x* by minimizing the *Gram loss*, defined as a weighted sum of squared L2 distances between corresponding Gram matrices across layers:

(2) $$\begin{array}{c} L\left(\hat{x},x\right)=\sum_{l}\frac{1}{4M_{l}N_{l}^{2}}\;{∥{\hat{G}}^{l}-G^{l}∥}_{2}^{2}.\; \end{array}$$

where *N*_*l*_, is the number of features in layer *l*, and *M*_*l*_ is the number of units within each feature (note that the term $1/4M_{l}N_{l}^{2}$ used here to weight the contribution from each individual layer is not identical to the one in Gatys et al. 2015; see the Supplementary Information for details on our choice of loss weighting for image synthesis). To minimize the Gram loss, we used L-BFGS as in Gatys et al. (2015), using the implementation of the optimization algorithm provided by PyTorch (Ansel et al., 2024).

Minimizing this objective in pixel space generates novel images whose texture statistics, as captured by feature correlations in a convolutional neural network, match those of the original image. Importantly, while spatial structure is not preserved, the resulting images retain perceptually salient texture properties, highlighting the role of feature-level statistics in texture representation.

### CNN layer selection

We implemented the texture representation and the synthesis algorithm described above using PyTorch (Ansel et al., 2024) for a range of CNN architectures trained for object classification with ImageNet-1K (see Table 1). The pretrained networks were sourced from TorchVision (TorchVision maintainers & contributors, 2016). The convolutional layers of the CNN originally used by Gatys et al. (2015) (VGG-19) have five different spatial scales, corresponding to the five consecutive “convolve-and-pool” blocks of that architecture. Gatys et al. (2015) showed that selecting just one layer for each of these spatial scales was enough to produce perceptually satisfying synthetic textures. Therefore, to ensure that the results of our analysis are comparable across different networks, we extracted feature maps from five layers from each architecture, selected in such a way that they are located at comparable relative depth across architectures; see Supplementary Tables S1 and S2 for details.

**Table 1.** Convolutional neural network architectures grouped by family.

**Family**	**Model**
	AlexNet (Krizhevsky, 2009)
DenseNet (Huang, Liu & Weinberger, 2017)	DenseNet-121DenseNet-169DenseNet-201
InceptionNet (Howard et al. 2017)	Inception-v3 (Szegedy, Vanhoucke, Ioffe, Shlens, & Wojna, 2016)
ResNet (He, Zhang, Ren, & Sun, 2016)	ResNet18ResNet34ResNet50ResNet101ResNet152
VGG (Simonyan & Zisserman, 2015)	VGG-16VGG-19

### Stimulus dataset

The Describable Textures Dataset (DTD) (Cimpoi et al., 2014) is our input dataset of choice. It consists of 5,640 images of naturalistic visual textures collected from the Internet, spanning a wide range of materials and surface appearances. The dataset is organized into 47 texture classes, each one containing 120 images. The image size ranges between 300 × 300 px and 640 × 640 px. We resized (scaled) every image to 224 × 224 px, in order to match the CNNs’ input spatial dimensions and to maintain a stable representation of the textures.

The class labels arise from a human annotation procedure based on the Texture Lexicon, a psycholinguistic study introduced in Bhushan, Rao, and Lohse (1997) with the goal of constructing a linguistically grounded vocabulary for describing visual textures. The Texture Lexicon identifies a set of texture-related adjectives commonly used by human observers (e.g., *blotchy*, *striped*, *bumpy*, *woven*) and organizes them according to perceptual relevance.

To construct DTD, the authors selected 47 representative texture terms from the Texture Lexicon and used them as labels in a large-scale annotation task involving human participants. Annotators were presented with images and asked to assign one or more texture attributes that best described the visual appearance of the image. Final class labels were determined by aggregating human responses and retaining images for which there was sufficient agreement across annotators. As a result, DTD encodes texture categories that reflect shared perceptual judgments rather than low-level image statistics or semantic object labels.

This annotation procedure makes DTD particularly suitable for studies of texture perception, as it provides a direct link between image-level texture statistics and perceptually meaningful texture descriptors grounded in human language and cognition. DTD treats textures as entities that can be categorized in a manner analogous to object images, effectively providing human-defined clusters of visual textures with a balanced distribution of images across classes. The richness and diversity of DTD thus constitute a perceptual and linguistic benchmark against which we can evaluate whether texture representations derived from CNNs in combination with the Gatys method are sufficient to preserve class-specific texture distinctions as defined by human perception.

### Clustering

We performed representation similarity analysis (RSA) (Kriegeskorte, Mur, & Bandettini, 2008) on the texture representations extracted with the Gram matrices on our pool of 12 CNNs. RSA was introduced in neuroscience as a method to compare object representations obtained with different means (e.g., functional magnetic resonance imaging vs. magnetoencephalography) or to compare biological and computational representations (e.g., brain activity vs. CNNs). It relies on representational dissimilarity matrices (RDMs), where each entry is the pairwise dissimilarity between responses to two stimuli. We employed RSA to verify whether the RDMs produced with Gram matrices extracted from DTD show a clustering pattern consistent with the original 47 classes. For each of the 12 CNNs, we obtained five RDMs — one for each of the convolutional layers analyzed — of dimensionality 5,640 × 5,640, where each entry is the distance among Gram matrices for one pair of images in DTD.

We tested whether Gram matrix–based texture representations capture the structure of the 47 texture classes in DTD by performing hierarchical clustering with Ward linkage function based on Euclidean distance, as implemented in scikit-learn (Pedregosa et al., 2011). Hierarchical clustering is an unsupervised technique that groups data points based on their similarity, producing a tree-like representation in which clusters are progressively merged according to their pairwise distances. This hierarchical organization provides insight into the intrinsic structure of the data without relying on class labels.

To evaluate the extent to which the learned texture representations recover the human-defined categories in DTD, we treated the dataset labels as a reference standard. Specifically, we fixed the number of clusters to *k* = 47, matching the number of texture classes in the original dataset, and allowed the clustering algorithm to organize the representations accordingly. This procedure enables a direct comparison between the automatically discovered clusters and the perceptual texture classes defined by human annotations.

### Measuring clustering quality

We quantified texture information carried in the representations within Gram matrices with mutual information (MI) (MacKay, 2003). In our analysis, MI was computed between the real 47 classes in DTD (*real classes*) and the 47 clusters found by hierarchical clustering (*found clusters*). If *X* and *Y* are random variables representing, respectively, the true label of an image (*real class*) and the label assigned to it by the clustering procedure (*found cluster*), MI is the reduction in uncertainty, quantified as entropy (*H*), in one of the two variables associated with learning the value of other:

$$\begin{array}{ccc} \;\mathrm{MI}(X:Y)\; & = & \;H(X)-H(X|Y)=H(Y)-H(Y|X)\\ & & =\sum_{x,y}p\left(x,y\right)\;\log\;\frac{p(x,y)}{p(x)\;p(y)} \end{array}$$

By construction,  MI (X:Y) is upper-bounded by the minimum between *H*(*X*) and *H*(*Y*). This can be seen by the expression above taking into account that *H*(*X*|*Y*) and *H*(*Y*|*X*) are always nonnegative. Therefore, considering that *Y* follows an unknown distribution where *found clusters* result from hierarchical clustering, and *X* is distributed uniformly among the 47 *real classes* of DTD, in our case, the MI is upper-bounded by the entropy of *X*:

(3) $$\begin{array}{ccc} {\mathrm{MI}}_{\max}\; & = & \;H\left(X\right)=-\sum_{i=1}^{47}p_{i}\log_{2}p_{i}\\ & & =\log_{2}47\approx 5.5\text{bits}\; \end{array}$$

All empirical estimates for mutual information were corrected for potential undersampling bias using the NSB method (Nemenman, Shafee, & Bialek, 2002), as implemented by the ndd Python package (Marsili, 2021).

We quantified MI between *real classes* and *found clusters* for the *found classes* distribution drawn from each of the five layers of the 12 CNNs. Therefore, for each model, we obtained five MI values.

### Brain-Score correlation

To assess the relationship between the goodness of a CNN as a model for biological object recognition and texture perception, we correlated the best MI value (i.e., the highest of the five MI values computed for each model) of each CNN to Brain-Score. Brain-Score is a composite of multiple behavioral and neural benchmarks that determine how similar artificial neural networks are to the object recognition mechanisms in the human and macaque brain (Schrimpf et al., 2018). In this study, we focus on the following Brain-Score components: *neural vision*, *behavior vision*, and *average vision*, as well as the region-specific neural predictability scores for *V1, V2, V4*, and *IT*. The *neural vision* score summarizes a model’s ability to predict neural responses across areas of the primate ventral visual stream, while *behavior vision* quantifies the alignment between model predictions and human behavioral performance on object recognition tasks. The *average vision* score provides an overall summary across neural and behavioral benchmarks. The region-specific scores (*V1, V2, V4, IT*) evaluate how well model representations predict neural activity in individual cortical areas involved in the hierarchical processing of visual information along the ventral stream. For each of the 12 models, we correlated the highest MI with the aforementioned Brain-Score values, which we report in Supplementary Table S3. We run a linear correlation with Pearson’s *p* and set α = 0.05.

## Results

### Representational similarity analysis

**Figure 1.**
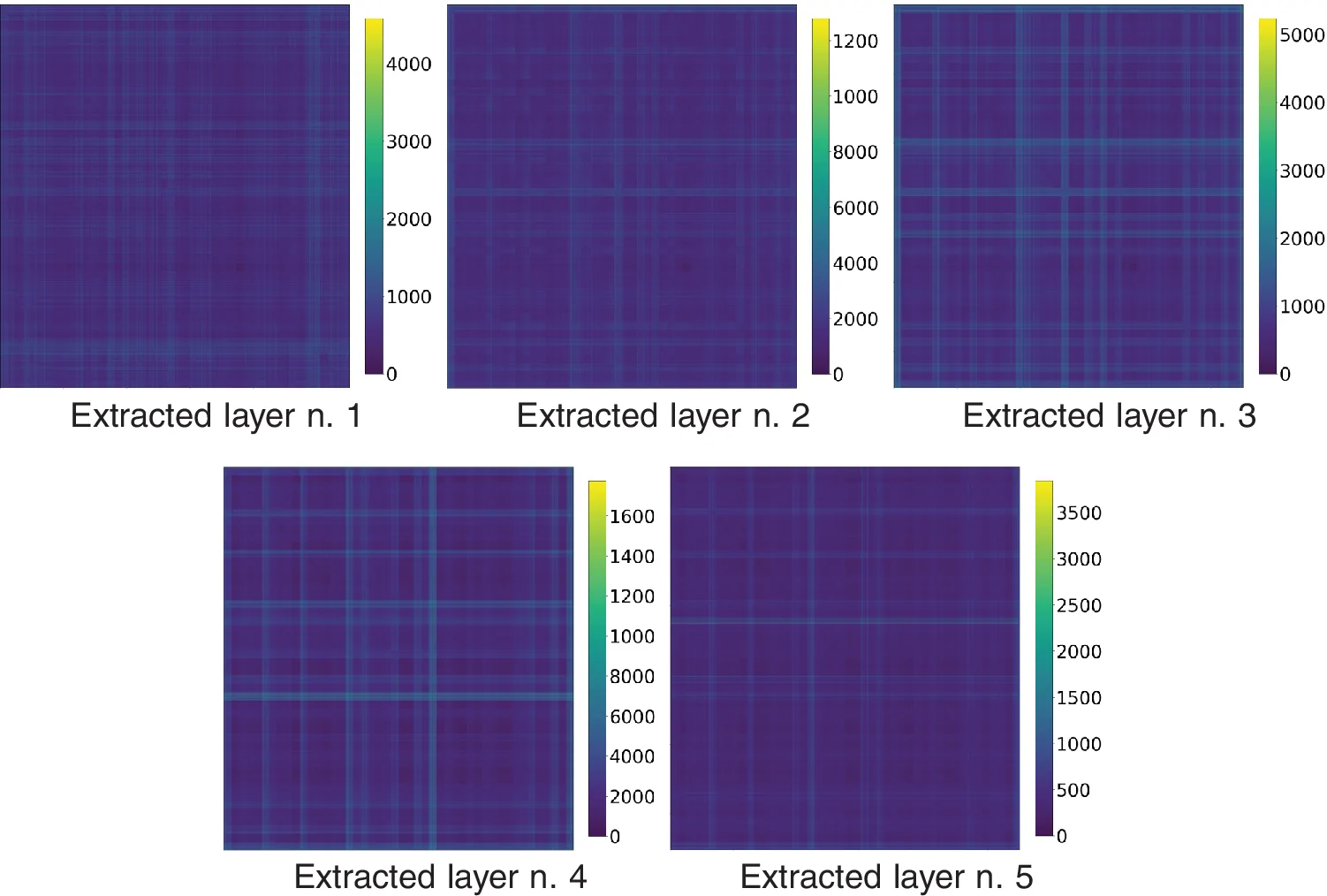
Representational dissimilarity matrices obtained from the five layers analyzed for VGG-19. The size of each matrix is 5,640 × 5,640, and each entry corresponds to the Euclidean distance — relative to each matrix, as in the color bars — between the Gram matrix representation of one pair of images in DTD. The entries are sorted such that images belonging to each of the 47 ground-truth categories are placed next to each other.

To understand how texture information is represented across the layers of our CNNs, we started by performing RSA. Figure 1 shows RDMs calculated on pairs of Gram matrix representations of DTD images extracted from VGG-19. Performing the same analysis on other networks led to qualitatively similar results (see de Paolis, 2025, for high-resolution RDM plots for all the networks considered in this work). Visual inspection reveals that the structure of the RDMs reflects the existence of at least some of the classes in DTD and that for this network, the clearest structure emerges in the last three layers. To understand these trends more quantitatively, we next moved to performing clustering analysis of the Gram representations.

### Clustering analysis

**Figure 2.**
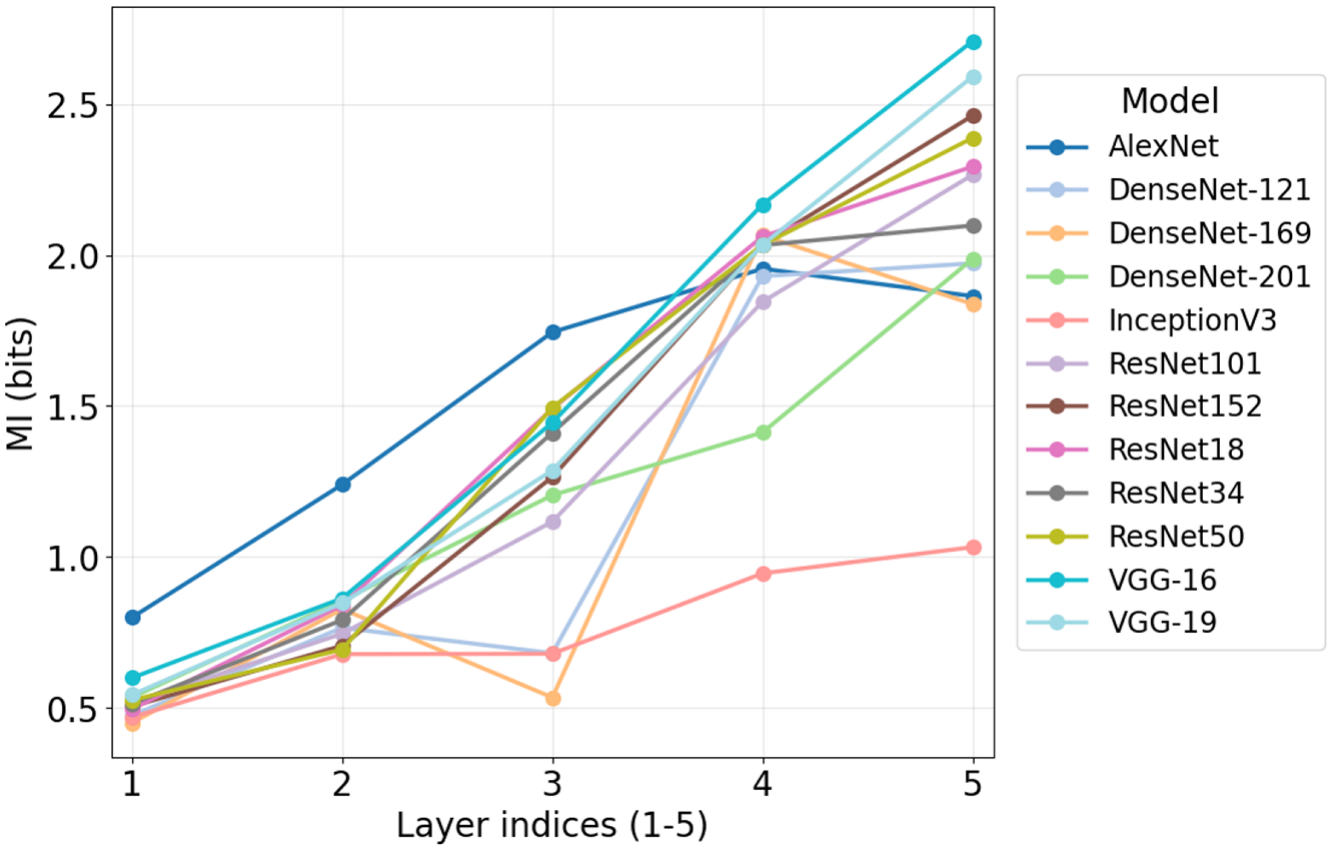
MI values across CNN layers identified by index (1–5). Each line corresponds to one of the 12 models as color-coded in the legend.

Figure 2 represents the values of MI by model and by layer found with hierarchical clustering. The plot shows a trend: MI grows along with the depth of the extracted features, consistently with the trend found by visual inspection in the RSA plots. The highest MI is yielded by the last layer for most models, but it does not go beyond ≈2.7 bits, which is about half of the maximum achievable MI, according to Equation 3. These numbers show that increasing the depth of the extracted representations leads to an increasing clustering quality.

**Figure 3.**
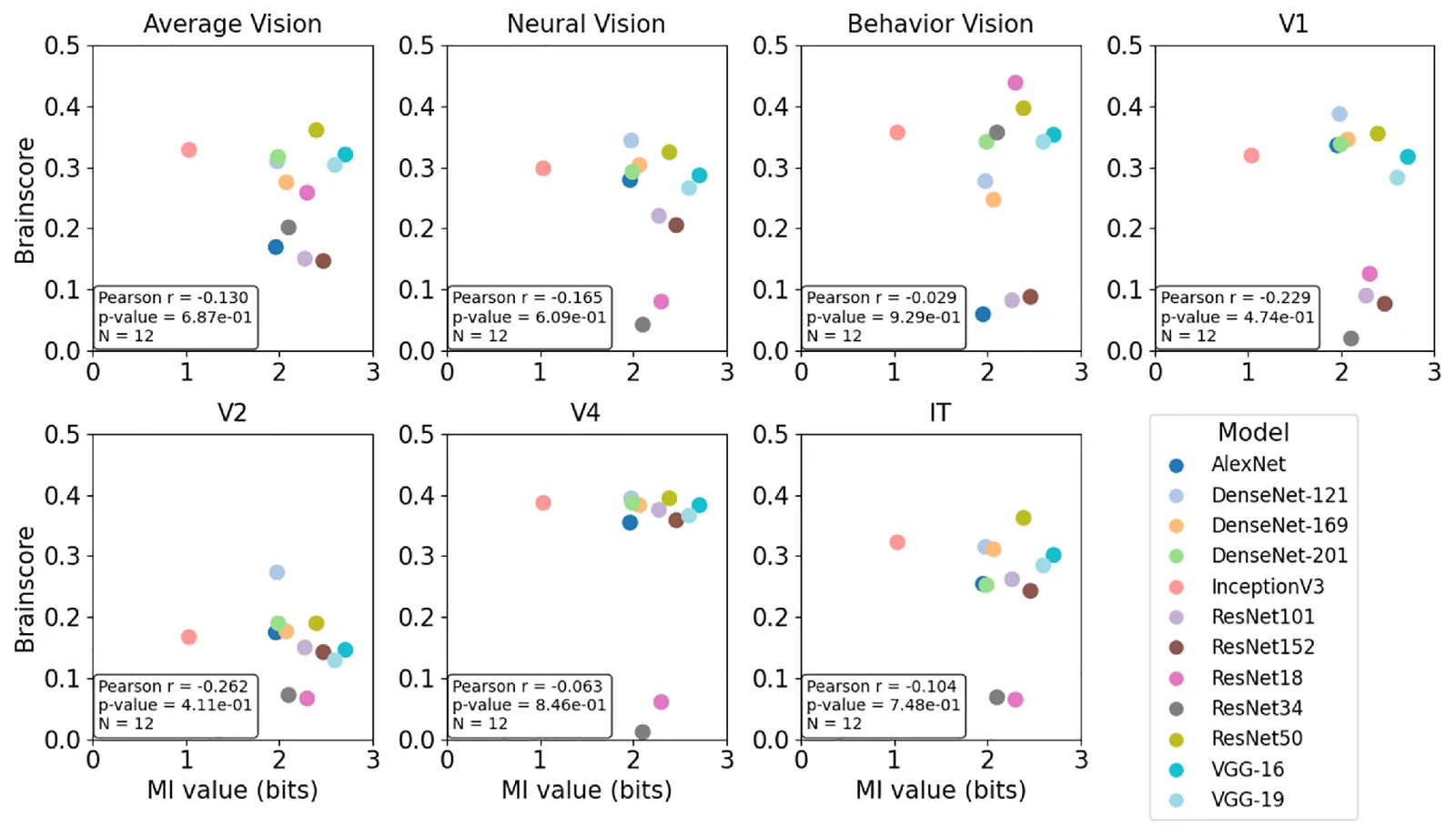
Plots of the correlations with Pearson’s *p*. Each subplot represents the correlation of Brain-Score benchmarks *average vision, neural vision, behavior vision, V1, V2, V4, IT*, respectively, vs. the layer with the highest MI for each of the 12 CNNs. Each color identifies a model as reported in the legend.

The results of the correlation among the highest MI value per model and selected Brain-Score benchmarks are reported in Table 2. We did not find any significant correlation between any Brain-Score value and the best MI found in any model, as shown in Figure 3.

**Table 2.** Correlation between texture representation quality, expressed as MI between the clusters in the Gram representation of textures and the ground-truth labels in DTD, and quality of the networks as models of the ventral visual stream, measured by Brain-Score. For more details on Brain-Score, see Brain-score correlation.

Brain-Score metric	Pearson’s *r*	*p*-value
Average vision	−0.130	0.68
Neural vision	−0.165	0.60
Behavior vision	−0.029	0.92
V1	−0.229	0.47
V2	−0.262	0.41
V4	−0.063	0.84
IT	−0.104	0.74

### Texture synthesis

**Figure 4.**
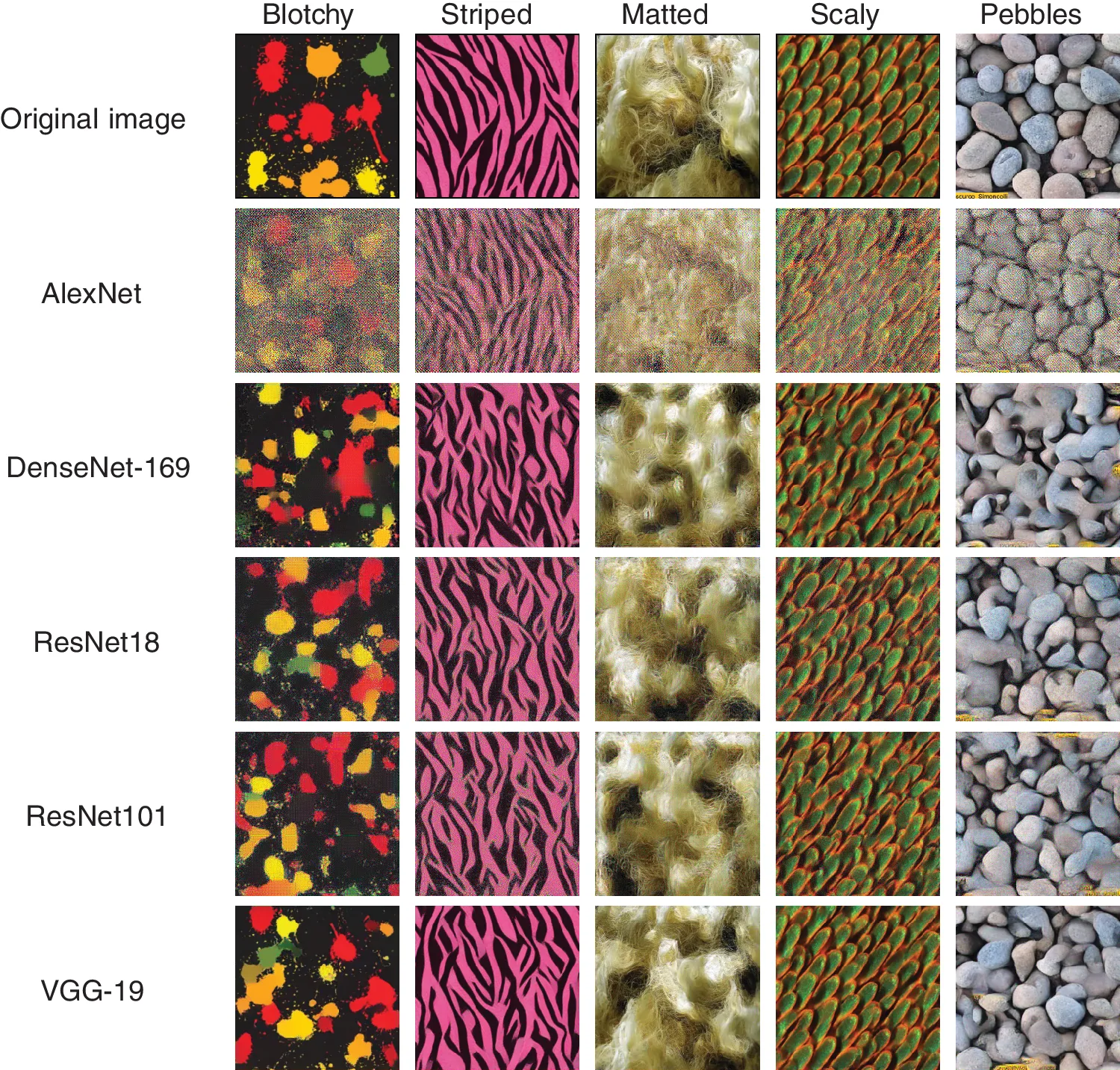
Textures generated with the Gatys algorithm applied to a subsample of CNNs (*rows*). The four original images (*top row*) belong to four different classes in DTD (columns: *blotchy, striped, matted, scaly*). The last column represents one of the images originally used by Gatys et al., which we synthesized as a reference. Textures generated by the whole pool of 12 models can be found in the (Supplementary Figures S1, S2).

In Figure 4, we report samples of synthesized textures for a subset of selected models. The purpose of this plot is to illustrate the output of the Gatys algorithm, as implemented by us with our pool of CNNs; we underline that texture synthesis is not part of the main analysis pipeline and, in our work, is only used to generate example visualizations. The results of the quantitative analyses described in the previous sections do not depend directly on this visual outcome. Each sample belongs to four separate DTD classes: *blotchy, striped, matted, scaly*. We observe how some models achieve a better perceptual output in terms of synthesized textures, despite running the same algorithm.

## Discussion

In this study, we have investigated the quality of the Gram texture representation proposed by Gatys et al. (2015) in terms of its agreement with the categorical grouping in the DTD (Cimpoi et al., 2014) for a diverse pool of deep convolutional networks. We have compared this measure of perceptual quality with Brain-Score, an independent benchmark for “brain-like” operation of the CNNs in object recognition settings (Schrimpf et al., 2018; Schrimpf et al., 2020).

Our analyses lead to three main conclusions. First, the perceptual quality of the Gram representations tends, for most networks, to grow monotonically with the depth of the layers analyzed (Figure 2). This is generally compatible with recent results suggesting that texture processing is distributed along intermediate areas of the ventral visual stream, including V2 and V4 (Okazawa et al., 2017; Ziemba et al., 2024) and possibly IT (Zhivago & Arun, 2014).

Second, the Gram representation is unable to recover the full class structure of the data, as determined by the human-assigned labels. Quantitatively, this is seen by the value of the clustering MI reaching only about half of the theoretical maximum (Figure 2). This highlights that, despite its popularity, the Gram representation is far from perfect. In further work, we aim at exploring whether the perceptual improvements associated with more sophisticated versions of the representation translate to significant improvements in MI on the DTD.

Third, the quality of the CNNs as implicit models for texture perception is not correlated with their quality as models of the ventral stream in an object recognition setting (Figure 3). This is despite the fact that there is substantial variability in both metrics, as is well known for Brain-Score, and as can be also appreciated visually for the textures in Figure 4. It is not simply due, for instance, to all models having roughly the same texture representation capacity. This result is surprising, because one could have expected that the functional features captured by Brain-Score are general enough to at least partly include those that are necessary to support human-like texture perception. Instead, this appears not to be the case. We conclude that in order to design methods of texture analysis and synthesis that are better aligned with human perception, we need to look beyond standard CNNs trained for object recognition, for instance, by incorporating unsupervised or self-supervised learning (Matteucci et al., 2024), multimodality (Radford et al., 2021), or different architectures, such as those with attention mechanisms (Guo, Deschaintre, Noll, & Roullier, 2022; Vaswani et al., 2017).

Finally, we note that our conclusions are broadly compatible with another series of works relating human vision and texture encoding in CNNs. In an influential paper, Geirhos et al. (2019) showed that CNNs trained for object classification on ImageNet tend to classify images based on texture information more than shape information, in a way that differs markedly from human behavior. However, subsequent work within that research strand points to the idea that, in fact, shape and texture information do coexist within the representation layers of CNNs and that this so-called texture bias emerges primarily at the level of the decision head, which, under standard training conditions, learns to prioritize texture information over shape information (Hermann, Chen, & Kornblith, 2020; Islam et al., 2021). Our notion of “texture information” (the MI between the Gram clusters and the ground-truth DTD labels) increases with layer depth across most models, generalizing the observations in Hermann, Chen, and Kornblith (2020), which shows (Supplementary Figure S1) that for AlexNet, the texture information increases rapidly in early convolutional layers and then flattens out toward the end of the hierarchy. This is very similar to the trend we report for AlexNet in Figure 2. Another interesting observation in Hermann, Chen, and Kornblith (2020) is that neurally motivated architectures like CorNET (Kubilius et al., 2018; designed by the same team as Brain-Score) do not fare better than standard CNNs from the point of view of their texture bias in classification. This echoes our own results showing that architectures that are better models of ventral stream processing in the canonical sense (related to object identity processing, measured by Brain-Score) are not necessarily more human-like in how they process textures.

## Conclusions

Our work highlights a number of limitations of popular machine learning–based approaches for visual texture analysis and synthesis. At the same time, it provides a simple example of a visual function that is likely performed in the ventral stream but for which the existing quality metrics of neural and behavioral fidelity for models seem to have very little predictive power. In future work, we plan to extend our analyses to different architectures and texture representations, as well as develop a more direct and systematic way of testing the perceptual quality of generated textures with large-scale human psychophysics. Going forward, we hope that our approach can provide an additional guide for the development of truly general-purpose models of the mammalian visual system.

## Supplementary Material

Supplement 1
