# Supplementary material for: Perceptual misalignment of texture representations in convolutional neural networks
Source: J Vis. 2026 Sep 18;26(9):9. doi: 10.1167/jov.26.9.9 (PMC13614142; doi:10.1167/jov.26.9.9)
Supplement: Supplementary file 1 [file jovi-26-9-9_s001.pdf]

Perceptual misalignment of texture representations in convolutional neural networks

Supplementary Information

Ludovica de Paolis[1], Fabio Anselmi[2], Alessio Ansuini[3], and Eugenio Piasini[1]

[1]Neuroscience area, International School for Advanced Studies (SISSA), Trieste, Italy

[2]Department of Mathematics, Informatics and Geosciences, Università degli Studi di Trieste, Trieste, Italy

[3]Department of Data Engineering, Area Science Park, Trieste, Italy

1 Loss normalization

In this section we derive the weighting rule we used to combine the Gram losses from multiple layers.

Consider the Gram matrix G for a certain layer l. Its (i, j)-th entry is

$$G_{ij} = \sum_{n=1}^{M} F_i^n F_j^n$$

where the index n runs over all elements of the i-th feature map F_i (so for instance in a typical convolutional network M would be the product of the height and width of the feature map). Assume that the individual activations of the network units are roughly

normally distributed:

$$F_i^n \sim \mathcal{N}(0,1)$$

This is a reasonable assumption in presence of batch normalization. Our goal is now to understand how the "Gram loss" for this layer is distributed. The Gram loss is

$$\mathcal{L}_l = \sum_{i=1}^{N} \sum_{j=1}^{N} \left(G_{ij}^{\text{rec}} - G_{ij}^{\text{orig}} \right)^2$$

For a fixed value of i, j and n let's define two new random variables A and B:

$$A = \left(\frac{F_i^n + F_j^n}{\sqrt{2}} \right)^2 \quad , \quad B = \left(\frac{F_i^n - F_j^n}{\sqrt{2}} \right)^2$$

from which we get

$$\frac{A-B}{2} = \frac{1}{2} \left[\frac{F_i^2 + F_j^2 + 2F_iF_j}{2} - \frac{F_i^2 + F_j^2 - 2F_iF_j}{2} \right] = \frac{1}{4} \left[4F_iFj \right] = F_iF_j$$

so we can think of G_{ij} as the sum of M iid terms, each of which is distributed like $(A-B)/2$. By observing that $(F_i^n + F_j^n)/\sqrt{2} \sim \mathcal{N}(0,1)$, we see that A and B are each simply the square of a standard Normal. Therefore they both follow a Chi-squared distribution with one degree of freedom

$$A \sim \chi_1^2 \quad , \quad B \sim \chi_1^2$$

which means that

$$\mathbb{E}[A] = \mathbb{E}[B] = 1 \quad , \quad \text{Var}[A] = \text{Var}[B] = 2$$

Therefore, $A - B$ is the difference of two independent random variables with mean zero

and variance 2. This implies that, for any n,

$$\mathbb{E}[2F_i^n F_j^n] = \mathbb{E}[A - B] = 0$$
$$\mathrm{Var}[2F_i^n F_j^n] = \mathrm{Var}[A] + \mathrm{Var}[B] = 4$$

and therefore

$$\mathbb{E}[F_i^n F_j^n] = 0$$
$$\mathrm{Var}[F_i^n F_j^n] = 2$$

So G_{ij} is the sum of a large number M of iid random variables, each with mean 0 and variance 2. By the central limit theorem, then,

$$G_{ij} \sim \mathcal{N}\left(0, \sqrt{2M}\right)$$

If this is true for both the original and reconstructed Gram, then

$$G_{ij}^{\mathrm{rec}} - G_{ij}^{\mathrm{orig}} \sim \mathcal{N}(0, 2\sqrt{M})$$

and therefore

$$\frac{G_{ij}^{\mathrm{rec}} - G_{ij}^{\mathrm{orig}}}{2\sqrt{M}} \sim \mathcal{N}(0,1)$$

Now, consider the quantity

$$\frac{\mathcal{L}_l}{4M} = \sum_{i=1}^{N}\sum_{j=1}^{N}\left(\frac{G_{ij}^{\mathrm{rec}} - G_{ij}^{\mathrm{orig}}}{2\sqrt{M}}\right)^2$$

It is clear that $\mathcal{L}_l/4M$ is the sum of N^2 squared standard Normal random variables. Therefore,

$$\frac{\mathcal{L}_l}{4M} \sim \chi^2_{N^2}$$

and as a consequence

$$\mathbb{E}\left[\frac{\mathcal{L}_l}{4M}\right] = N^2$$

Finally, this implies that

$$\mathbb{E}\left[\mathcal{L}^l\right] = 4MN^2$$

In other words, if the activations F_i^n are standardized, the expected value of the Gram loss for a layer with N features of size M is $4MN^2$, just as a consequence of the size and shape of the layer. Therefore, to make sure that Gram losses coming from different layers have the same weight in our total loss we should divide each layer-specific Gram loss by $4MN^2$, where M and N are the values specific to that layer.

2 Supplementary Tables

Supplementary Table 1: Extracted layers from each CNN and relative Gram matrix dimensionality. The names refer to the terminology of the models' versions in Torchvision. For models whose layers are not associated with a name, we report the absolute index.

**Model**	**Extracted layers**	**Gram dimensionality**
AlexNet	`layer idx=[0, 3, 6, 8, 10]`	L0=$64(64+1)/2 = 2080$<br>L3=$192(192+1)/2 = 18520$<br>L6=$384(384+1)/2 = 73920$<br>L8=$256(256+1)/2 = 32896$<br>L10=$256(256+1)/2 = 32896$
DenseNet-121	`bn_0=features.norm0`<br>`bn_1=features.denseblock1.denselayer1.norm2`<br>`bn_2=features.denseblock2.denselayer1.norm2`<br>`bn_3=features.denseblock3.denselayer1.norm2`<br>`bn_4=features.denseblock4.denselayer1.norm2`	bn0=$64(64+1)/2 = 2080$<br>bn1=$128(128+1)/2 = 8256$<br>bn2=$128(128+1)/2 = 8256$<br>bn3=$128(128+1)/2 = 8256$<br>bn4=$128(128+1)/2 = 8256$
DenseNet-169	`bn_0=features.norm0`<br>`bn_1=features.denseblock1.denselayer1.norm2`<br>`bn_2=features.denseblock2.denselayer1.norm2`<br>`bn_3=features.denseblock3.denselayer1.norm2`<br>`bn_4=features.denseblock4.denselayer1.norm2`	bn0=$64(64+1)/2 = 2080$<br>bn1=$128(128+1)/2 = 8256$<br>bn2=$128(128+1)/2 = 8256$<br>bn3=$128(128+1)/2 = 8256$<br>bn4=$128(128+1)/2 = 8256$
DenseNet-201	`bn_0=features.norm0`<br>`bn_1=features.denseblock1.denselayer1.norm2`<br>`bn_2=features.denseblock2.denselayer1.norm2`<br>`bn_3=features.denseblock3.denselayer1.norm2`<br>`bn_4=features.denseblock4.denselayer1.norm2`	bn0=$64(64+1)/2 = 2080$<br>bn1=$128(128+1)/2 = 8256$<br>bn2=$128(128+1)/2 = 8256$<br>bn3=$128(128+1)/2 = 8256$<br>bn4=$128(128+1)/2 = 8256$

Supplementary Table 2: (Continued from previous page)

**Model**	**Extracted layers**	Gram matrix dimensionality
Inception-v3	`bn_0=Conv2d_1a_3x3.bn`	bn0=$32(32+1)/2=561$
	`bn_1=Conv2d_2a_3x3.bn`	bn1=$32(32+1)/2=561$
	`bn_2=Conv2d_2b_3x3.bn`	bn2=$64(64+1)/2=2080$
	`bn_3=Conv2d_3b_1x1.bn`	bn3=$80(80+1)/2=3240$
	`bn_4=Conv2d_4a_3x3.bn`	bn4=$192(192+1)/2=185208$
ResNet18	`bn_0=layer1.0.bn1`	bn0=$64(64+1)/2=2080$
	`bn_1=layer1.0.bn1`	bn1=$64(64+1)/2=2080$
	`bn_2=layer2.0.bn1`	bn2=$128(128+1)/2=8256$
	`bn_3=layer3.0.bn1`	bn3=$256(256+1)/2=32896$
	`bn_4=layer4.0.bn1`	bn4=$512(215+1)/2=131280$
ResNet34	`bn_0=layer1.0.bn1`	bn0=$64(64+1)/2=2080$
	`bn_1=layer1.0.bn1`	bn1=$64(64+1)/2=2080$
	`bn_2=layer2.0.bn1`	bn2=$128(128+1)/2=8256$
	`bn_3=layer3.0.bn1`	bn3=$256(256+1)/2=32896$
	`bn_4=layer4.0.bn1`	bn4=$512(215+1)/2=131280$
ResNet50	`bn_0=layer1.0.bn1`	bn0=$64(64+1)/2=2080$
	`bn_1=layer1.0.bn1`	bn1=$64(64+1)/2=2080$
	`bn_2=layer2.0.bn1`	bn2=$128(128+1)/2=8256$
	`bn_3=layer3.0.bn1`	bn3=$256(256+1)/2=32896$
	`bn_4=layer4.0.bn1`	bn4=$512(215+1)/2=131280$
ResNet101	`bn_0=layer1.0.bn1`	bn0=$64(64+1)/2=2080$
	`bn_1=layer1.0.bn1`	bn1=$64(64+1)/2=2080$
	`bn_2=layer2.0.bn1`	bn2=$128(128+1)/2=8256$
	`bn_3=layer3.0.bn1`	bn3=$256(256+1)/2=32896$
	`bn_4=layer4.0.bn1`	bn4=$512(215+1)/2=131280$
ResNet152	`bn_0=layer1.0.bn1`	bn0=$64(64+1)/2=2080$
	`bn_1=layer1.0.bn1`	bn1=$64(64+1)/2=2080$
	`bn_2=layer2.0.bn1`	bn2=$128(128+1)/2=8256$
	`bn_3=layer3.0.bn1`	bn3=$256(256+1)/2=32896$
	`bn_4=layer4.0.bn1`	bn4=$512(215+1)/2=131280$
VGG-16	`bn idx=[1, 8, 15, 25, 35]`	bn1=$64(64+1)/2=2080$
		bn8=$128(128+1)/2=8256$
		bn15=$256(256+1)/2=32896$
		bn25=$512(215+1)/2=131280$
		bn35=$512(215+1)/2=131280$
VGG-19	`bn idx=[1, 8, 15, 28, 41]`	bn1=$64(64+1)/2=2080$
		bn8=$128(128+1)/2=8256$
		bn15=$256(256+1)/2=32896$
		bn25=$512(215+1)/2=131280$
		bn35=$512(215+1)/2=131280$

Supplementary Table 3: Summary of the values from Brain-Score benchmarks that we selected for our study (columns), for the 12 CNNs from our pool (rows). In the second column and second row, for models and benchmarks respectively, we report the .csv coordinates of the leaderboard. Our Brain-Score values were exported from the leaderboard website on September 8th 2025.

**Model**	**Model coordinates**	**average vision**	**neural vision**	**behavior vision**	**V1**	**V2**	**V4**	**IT**
**Benchmark coordinates**		D2	E2	DB2	F2	AM2	AP2	AV2
**AlexNet**	B290	0.171	0.281	0.06	0.338	0.176	0.355	0.255
**Densenet-121**	B128	0.311	**0.344**	0.278	**0.388**	**0.275**	**0.396**	0.315
**DenseNet-169**	B158	0.276	0.305	0.248	0.346	0.178	0.384	0.312
**DenseNet-201**	B94	0.318	0.293	0.343	0.34	0.19	0.388	0.253
**Inception-v3**	B84	0.33	0.3	0.358	0.321	0.169	**0.388**	0.324
**Resnet18**	B354	0.152	0.221	0.084	0.091	0.151	0.377	0.263
**Resnet34**	B357	0.147	0.206	0.089	0.077	0.143	0.359	0.244
**Resnet50**	B60	**0.362**	0.326	**0.397**	0.356	0.191	0.395	**0.363**
**Resnet101**	B176	0.26	0.081	0.44	0.127	0.068	0.062	0.066
**Resnet152**	B228	0.202	0.044	0.359	0.021	0.074	0.012	0.069
**VGG-16**	B104	0.322	0.288	0.354	0.319	0.147	0.385	0.303
**VGG-19**	B148	0.305	0.267	0.343	0.285	0.13	0.367	0.285

3 Supplementary Figures

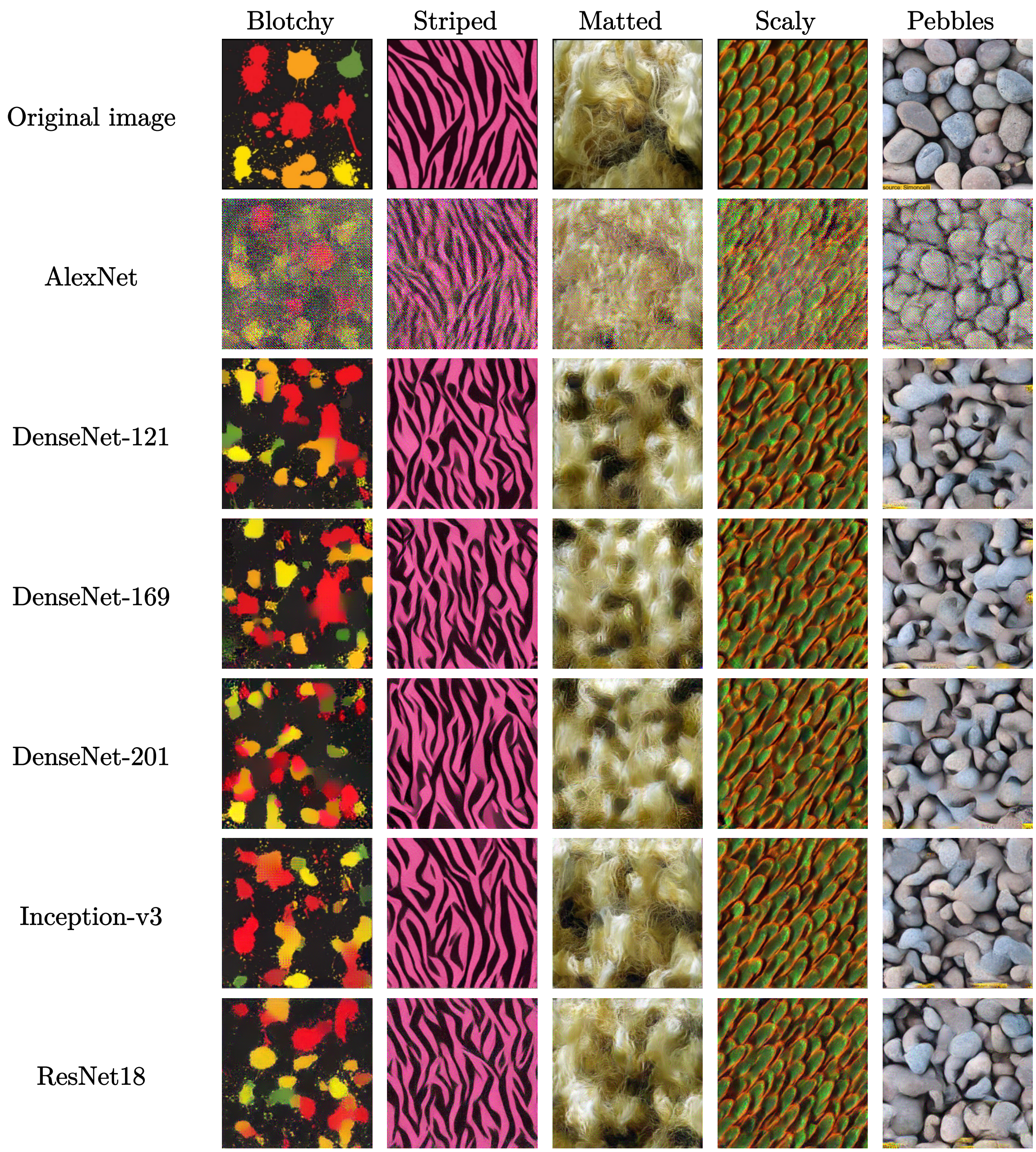

Supplementary Figure 1: Textures generated with the Gatys algorithm with the 12 CNNs of choice (rows). The 4 original images (top row) belong to 4 different classes in DTD (columns: *blotchy; striped; matted; scaly*). The last column represents one of the images originally used by Gatys et al., which we synthesized as a reference.

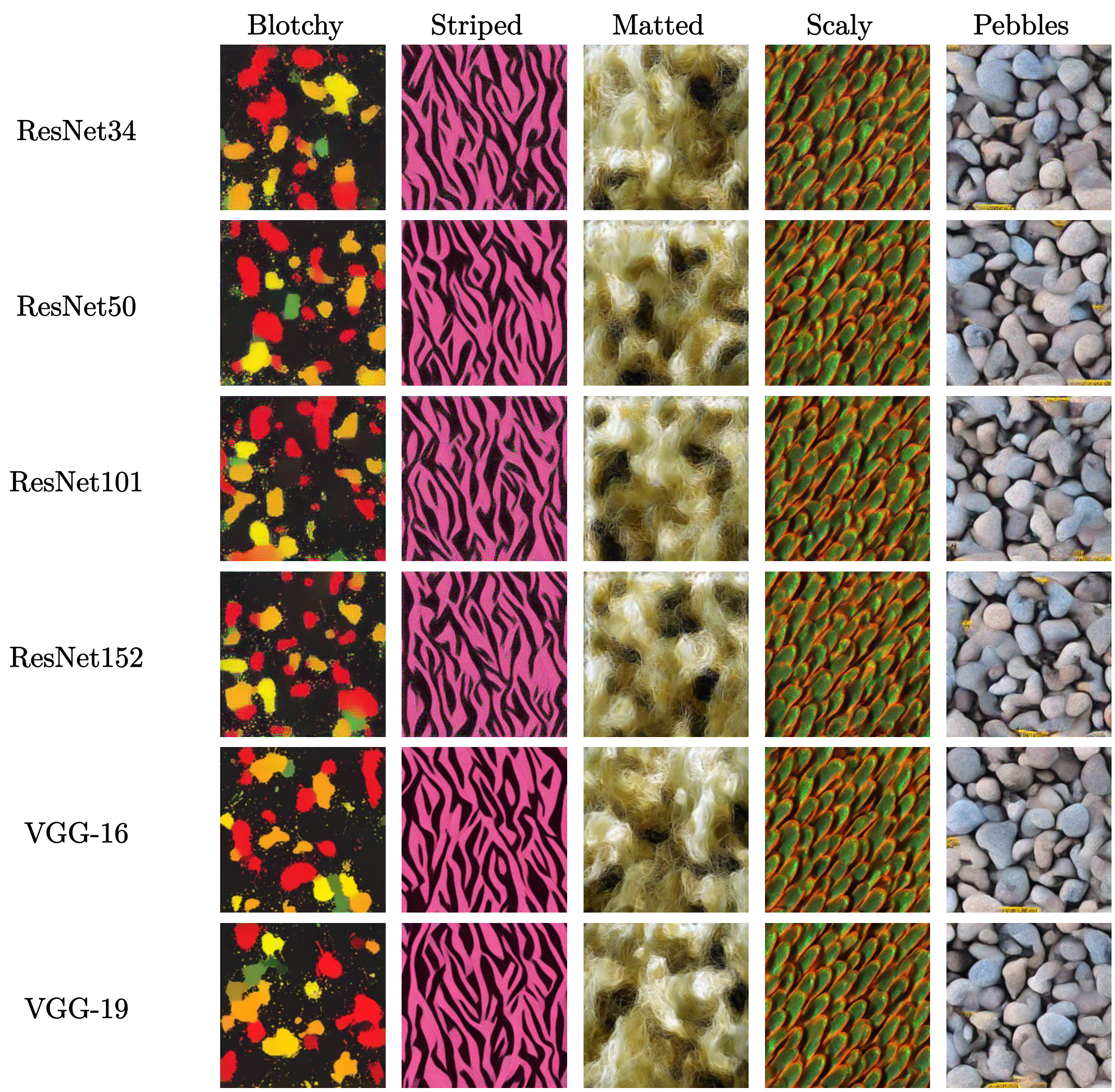

Supplementary Figure 2: (continued from previous page)
